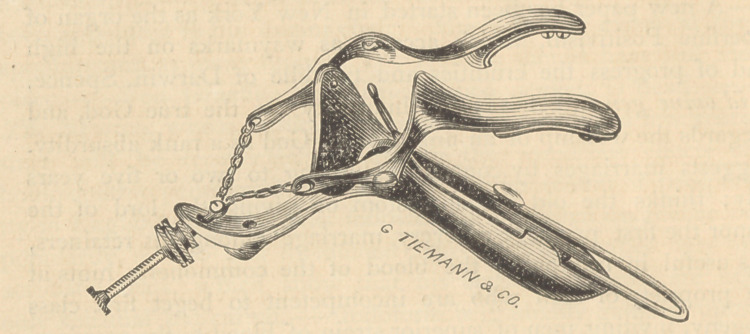# Loot

**Published:** 1870-09

**Authors:** 


### Loot.

***Nott's Improved Speculum.***

By the kindness of Messrs. Bliss & Sharp we are enabled to present an electrotype of this instrument for which the inventor claims the following advantages:

"1. No instrument hitherto devised can be more easy to introduce.

2. It can be equally well used in the semi-prone position or on the back.

3. While elevating or depressing the perineum, its feet are so constructed as to expand the *ostium vaginæ* to any desired extent.

4. It is perfectly self-retaining, without any arrangement external to the vagina.

5. In the semi-prone position it has the same advantage of atmospheric pressure as the lever speculum of Sims, and when the patient is on the back, by elevating the hips with a cushion or pillow, you have the same advantage of atmospheric pressure.

6. I use the instrument almost entirely with the patient on the back, because I can do everything I wish to do with more facility; and because the light from any window is more easily commanded. The concave surface of the speculum looking upwards, catches and throws the light fully on the anterior wall of the vagina and os uteri.

7. For all ordinary manipulations, where no cutting is required, instead of a table, any common bedstead or couch will command the light sufficiently from almost any window to give a good view. Baker Brown, in his operations for vesico-vaginal fistula, while using Sims' speculum, places the patient in the lithotomy position.

8. Like Sim's speculum, mine does not stretch the vagina *longitudinally*, and therefore allows the os uteri to be drawn down with a tenaculum near to the vulva.

9. The anterior wall of the vagina being left free, more space is afforded for operations.

10. With this speculum there are few operations that cannot be easily performed without an assistant.

I have also added, in some of the instruments, a small tenaculum, two inches long, with a little chain, any link of which may be made to catch on to a knob at the heel of the instrument. With a pair of forceps the tenaculum is fastened into the anterior lip of the uterus, and then drawn out and fixed at any point we desire.

I should remark that I have not yet tried this instrument in a case of vesico-vaginal fistula, and do not think it would answer well in any but small openings. By shortening the feet of the instrument, however, they would be out of the way, and the instrument would still be self-retaining. The feet need only be long enough to curve around the rami of the pubes, and thus not press upon or stretch the bladder; with this alteration I see no reason why it should not answer well for vesico-vaginal fistula."

Several of our friends who have tried it, think this the best speculum in use.

### *Is Syphilization to be Recommended for General Adoption?*

We extract the following from an able article, by Freeman J. Bumstead, in the *American Journal of Medical Sciences:*

"In the preceding pages I have said much in favor of this method of treatment, and I have endeavored to give it its full credit. From what I have personally witnessed, and from the accounts of others, I believe that it is a very effective method for the treatment of syphilis. I cannot say that I am fully convinced of the very small number of relapses after 'syphilization' alleged by its advocates; not that I for a moment doubt their honesty, but results so favorable as this should be confirmed by others less enthusiastic, and less interested, before demanding implicit belief. Should further examination and experiment show that only twelve or fourteen persons out of every hundred infected with syphilis and treated by repeated inoculations, ever exhibit any return of the disease, this method will have established very high claims in the treatment of syphilis, whenever circumstances will permit its being carried out, as it may be in our hospitals and other eleemosynary institutions.

"But, judging from what I have seen of the practice, nothing less than a very strong probability, in case I myself had syphilis, that the disease, if left alone, or if treated by mercury, would terminate disastrously, could induce me to undergo the personal discomfort, and for the length of time, which I have witnessed in the patients at Charity Hospital.

"This *debit* side of the account, I cannot believe, is fully appreciated by the advocates of 'syphilization' in their enthusiasm for the *credit*. The former, in fact, is apparently not regarded by them as deserving of mention. Upon inquiry of Prof. Boeck, I am told that this plan of treatment is usually carried on without any interference with the patient's ordinary avocations; that the inconvenience is even so light, that a husband or a wife who has gone astray and contracted syphilis, may undergo this series of repeated inoculations extending over a period of three or four months, and yet be able to pass off the resulting pustules and ulcerations, covering the chest, arms and thighs, as common 'boils!'

"What I have seen of 'syphilization,' as practiced by Prof. Boeck himself, would make it appear a less agreeable process than the above statement would imply. To be sure, the treatment was new at Charity Hospital, and the patients were probably aware of the fact and more or less suspicious. Yet they kept their beds during the greater part of the three or four months that the inoculations were going on, although they had every inducement to be up and out upon the grounds; and it often required all our power of persuasion to lead them to consent to a continuance of the inoculations, so great was their discontent. Indeed, I never made a visit to the hospital without the fear that some of them had eloped, as actually happened in three instances. They represented that the soreness of the ulcerations was so great that they could scarcely endure the contact of the bedclothes, much less that of their daily dress, and the appearance of the sores corroborated their statement. I cannot well imagine how persons in their condition could have been about attending to their daily business. When they left the hospital they bore scars over the chest, arms and thighs, which they will doubtless carry with them to their graves. Moreover, the serious tendency of an ulcer upon the thigh, in the case of Benner, to take on phagedenic action, shows that this practice is not devoid of danger. In short, I feel obliged to subscribe to the opinion expressed by Messrs. Lane and Gascoyen, that 'syphilization is not a treatment which can be recommended for adoption.'"

Practically, the facts seem to be that patients will get better even when they are covered with sores from this absurd practice, provided the destructive medication which it supercedes is followed also by generous fare and attention to the great requirements of physiological therapeutics. Nature will eliminate very successfully in the majority of instances; the poison itself is a sufficient stimulus of disintegration, and a wholesome, nutritious diet will energize both, and afford at the same time new and healthy tissue. The inoculation of the virus, fortunately, as a therapeutic, rarely produces sores serious enough to materially impede the case, when the proper rules of hygiene are observed.

***A Simple, Cheap and Efficient Substitute for the Stomach Pump.*** By John T. Hodgen, M.D., Professor of Anatomy, St. Louis Medical College.

About a year ago, I had a case of stricture of the œsophagus so narrow that my patient could not swallow even liquids. To sustain life I resorted to a small stomach tube (a gum catheter, in fact), as a means of injecting liquid nourishment; to this I fixed the elastic tube of one of Davidson's syringes.

On one occasion the vessel containing the liquid happened to be higher than the patient's stomach, and I observed while the syringe was not being used, that the fluid continued to flow into the stomach—the action being that of a syphon. I at once, to test the syphon, substituted a simple elastic tube for the syringe, and found the stomach could be as readily emptied as filled. Thus I conceived the idea of using a syphon instead of a stomach pump, and have used the same in a case of poisoning recently with the most complete success.

I attach four feet of India rubber tubing to a stomach tube, fill both with water by simply dipping it in the liquid end first, then compressing the elastic tube between the thumb and finger to keep the fluid from running out, introduce the stomach tube, lower the outer end of the elastic tube, and the contents of the stomach pour out as readily as if from an open vessel. When the fluid ceases to flow, I dip the outer end of the tube beneath the surface of water, elevate the vessel containing it, and the stomach is soon filled; lower again the outer end of the tube, and the stomach is emptied. This can, of course, be repeated as often as is necessary.

The advantages claimed for this simple contrivance are, that it may be almost improvised, is of speedy and easy application, has no valves to become obstructed or deranged, and is less expensive than a stomach pump.

The same principle may be applied in injecting fluids into the bowels, as indeed it has been for injecting into the bladder, uterus and vagina.—*Saint Louis Medical and Surgical Journal.*

In the *American Medical Recorder*, July, 1823, p. 583, Dr. Alexander Somervail, of Essex Co., Virginia, thus discourses:

"Take a flexible tube of proper size, and four feet long, one end of which prepared for passing into the stomach, and the other terminating in a funnel. When this is introduced into the stomach, water may be poured into the funnel, while the tube is kept perpendicular its whole length; by which means the stomach may be filled as an hydrostatic bellows; when this is accomplished, and the funnel full, if this latter is quickly turned down, so as to remain as low as its length will allow, all the liquid will run out again, as it will then be converted into a syphon. In this way it is believed the stomach may be filled and emptied as often as water can be poured through the funnel and tube into it.

"Perhaps in some cases the force of the syringe may be necessary in order to agitate the contents of the stomach, and mix it with the water, but in case of laudanum and spirits, these will mix with the water as readily as they would in a glass."

Which should suggest to Prof. Hodgen that "there is nothing new under the sun"—except Hydrate of Chloral.

The stomach pump itself was practically introduced to the profession by Prof. Physick in 1800, although claimed over twenty years afterwards by an English surgeon, Jukes, as a new invention of his own. It appears, however, that the idea was suggested by Dr. Alexander Munro, Jr., of Edinburgh, in his inaugural thesis in 1797, a fact of which Prof. Physick was ignorant until many years afterwards, when Dr. Munro incidentally mentioned it in his book on Morbid Anatomy.

***Hydrate of Chloral.***

Mr. F. E. Clarke extols (*Lancet*, April 16, 1870,) chloral as a palliative in malignant diseases. He regards it as the best palliative in malignant disease. He regards it as the best palliative in cancer for alleviating pain, and by its beneficial effects it enables the constitution to hold out longer against the ravages of the disease, and "thereby afforded a much greater chance of spontaneous cure, rare instances of which occur by the sloughing of the entire mass."

Mr. Weeden Cooke, Surgeon to the Cancer Hospital, also bears strong testimony (*Lancet*, April 30, 1870) to the value of chloral for the relief of pain in cancer, and its superiority over other means hitherto employed for that purpose. As a night draught he has found twenty grains quite sufficient, but when the pain is persistent, ten-grain doses, three times a day, give the greatest satisfaction. There is no headache, no sickness, no loss of appetite, nothing to hinder the patient taking exercise, and, so far as the disease will permit, pursuing his usual vocation.

## Figures and Tables

**Figure f1:**